# 1-Allyl-4-phenyl-2,3-dihydro-1*H*-1,5-benzodiazepin-2-one

**DOI:** 10.1107/S1600536810016004

**Published:** 2010-05-08

**Authors:** Daouda Ballo, Noureddine Hamou Ahabchane, Hafid Zouihri, El Mokhtar Essassi, Seik Weng Ng

**Affiliations:** a Laboratoire de Chimie Organique Hétérocyclique, Pôle de Compétences Pharmacochimie, Université Mohammed V-Agdal, BP 1014 Avenue Ibn Batout, Rabat, Morocco; b CNRST Division UATRS, Angle Allal Fassi/FAR, BP 8027 Hay Riad, 0000 Rabat, Morocco; c Department of Chemistry, University of Malaya, 50603 Kuala Lumpur, Malaysia

## Abstract

The seven-membered ring in the title compound, C_18_H_16_N_2_O, adopts a boat conformation with the two phenyl­ene carbons representing the stern and the methyl­ene C atom the prow. The dihedral angle between the best plane through the seven-membered ring and the phenyl ring is 62.13 (3)°.

## Related literature

For the background information on benzodiazepines, see: Ahabchane *et al.* (1999 ▶).
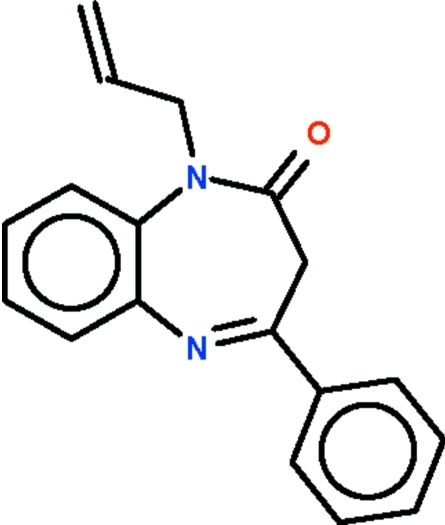


## Experimental

### 

#### Crystal data

C_18_H_16_N_2_O
*M* *_r_* = 276.33
Monoclinic, 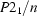

*a* = 11.4863 (3) Å
*b* = 6.0053 (2) Å
*c* = 20.3667 (5) Å
β = 93.525 (1)°
*V* = 1402.21 (7) Å^3^
*Z* = 4
Mo *K*α radiation
μ = 0.08 mm^−1^
*T* = 100 K
0.41 × 0.33 × 0.15 mm

#### Data collection

Bruker X8 APEX2 diffractometer
18220 measured reflections
4096 independent reflections
3417 reflections with *I* > 2σ(*I*)
*R*_int_ = 0.030

#### Refinement

*R*[*F* ^2^ > 2σ(*F* ^2^)] = 0.044
*wR*(*F* ^2^) = 0.153
*S* = 1.07
4096 reflections
190 parameters
H-atom parameters constrained
Δρ_max_ = 0.41 e Å^−3^
Δρ_min_ = −0.33 e Å^−3^

### 

Data collection: *APEX2* (Bruker, 2008 ▶); cell refinement: *SAINT* (Bruker, 2008 ▶); data reduction: *SAINT*; program(s) used to solve structure: *SHELXS97* (Sheldrick, 2008 ▶); program(s) used to refine structure: *SHELXL97* (Sheldrick, 2008 ▶); molecular graphics: *X-SEED* (Barbour, 2001 ▶); software used to prepare material for publication: *publCIF* (Westrip, 2010 ▶).

## Supplementary Material

Crystal structure: contains datablocks global, I. DOI: 10.1107/S1600536810016004/bt5257sup1.cif

Structure factors: contains datablocks I. DOI: 10.1107/S1600536810016004/bt5257Isup2.hkl

Additional supplementary materials:  crystallographic information; 3D view; checkCIF report

## Figures and Tables

**Fig. 1.**
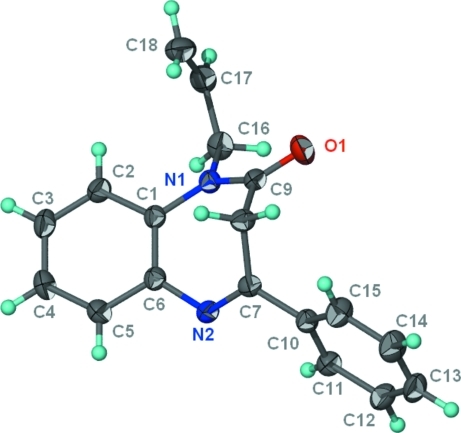
Anisotropic displacement ellipsoid plot (Barbour, 2001) of C18H16N2O at the 70% probability level; hydrogen atoms are drawn as spheres of arbitrary radius.

## supplementary crystallographic information

### Comment

The compound belongs to the class of benzodiazepine drugs; the background to this class of pharmaceutically potent compounds is explained in an earlier report (Ahabchane *et al.*, 1999). It is readily synthesized by reacting 4-phenyl-1,5-benzodiazepin-2-one with allyl bromide in the presence of a catalyst. The compound features a seven-membered ring fused with a phenylene ring (Scheme I, Fig. 1).

### Experimental

To a solution of 4-phenyl-1,5-benzodiazepin-2-one (1 g, 4.2 mmol) in DMF (20 ml) was added allyl bromide (0.5 g, 4.2 mmol), potassium carbonate (1 g, 7.4 mmol) and a catalytic quantity of tetra-*n-*butylammonium bromide. The mixture was stirred at room temperature for 24 h. The solution was filtered and the solvent removed under reduced pressure. The residue was recrystallized from ethanol to afford the title compound as colorless crystals.

### Refinement

H-atoms were placed in calculated positions (C—H 0.95–0.99 Å) and were included in the refinement in the riding model approximation, with *U*(H) set to 1.2*U*_eq_(C).

### Crystal data

**Table:**

C_18_H_16_N_2_O	*F*(000) = 584
*M**_r_* = 276.33	*D*_x_ = 1.309 Mg m^−^^3^
Monoclinic, *P*2_1_/*n*	Mo *K*α radiation, λ = 0.71073 Å
Hall symbol: -P 2yn	Cell parameters from 6537 reflections
*a* = 11.4863 (3) Å	θ = 3.4–32.8°
*b* = 6.0053 (2) Å	µ = 0.08 mm^−^^1^
*c* = 20.3667 (5) Å	*T* = 100 K
β = 93.525 (1)°	Block, colorless
*V* = 1402.21 (7) Å^3^	0.41 × 0.33 × 0.15 mm
*Z* = 4	

### Data collection

**Table:**

Bruker X8 APEX2 diffractometer	3417 reflections with *I* > 2σ(*I*)
Radiation source: fine-focus sealed tube	*R*_int_ = 0.030
graphite	θ_max_ = 30.0°, θ_min_ = 2.0°
φ and ω scans	*h* = −16→16
18220 measured reflections	*k* = −8→8
4096 independent reflections	*l* = −28→28

### Refinement

**Table:**

Refinement on *F*^2^	Primary atom site location: structure-invariant direct methods
Least-squares matrix: full	Secondary atom site location: difference Fourier map
*R*[*F*^2^ > 2σ(*F*^2^)] = 0.044	Hydrogen site location: inferred from neighbouring sites
*wR*(*F*^2^) = 0.153	H-atom parameters constrained
*S* = 1.07	*w* = 1/[σ^2^(*F*_o_^2^) + (0.0972*P*)^2^ + 0.2414*P*] where *P* = (*F*_o_^2^ + 2*F*_c_^2^)/3
4096 reflections	(Δ/σ)_max_ = 0.001
190 parameters	Δρ_max_ = 0.41 e Å^−^^3^
0 restraints	Δρ_min_ = −0.32 e Å^−^^3^

### Fractional atomic coordinates and isotropic or equivalent isotropic displacement parameters (Å^2^)

**Table:**

	*x*	*y*	*z*	*U*_iso_*/*U*_eq_	
O1	0.40991 (7)	0.27035 (17)	0.69505 (4)	0.0312 (2)	
N1	0.28667 (7)	0.50838 (16)	0.63865 (4)	0.0198 (2)	
N2	0.45689 (8)	0.81559 (16)	0.58166 (4)	0.0203 (2)	
C1	0.25876 (9)	0.64631 (18)	0.58323 (5)	0.0191 (2)	
C2	0.14324 (9)	0.6483 (2)	0.55638 (6)	0.0235 (2)	
H2	0.0875	0.5514	0.5738	0.028*	
C3	0.10918 (10)	0.7889 (2)	0.50490 (6)	0.0266 (3)	
H3	0.0308	0.7868	0.4870	0.032*	
C4	0.18956 (10)	0.9336 (2)	0.47934 (6)	0.0265 (3)	
H4	0.1664	1.0306	0.4441	0.032*	
C5	0.30340 (10)	0.9351 (2)	0.50565 (5)	0.0235 (2)	
H5	0.3577	1.0356	0.4885	0.028*	
C6	0.34063 (9)	0.79147 (18)	0.55714 (5)	0.0194 (2)	
C7	0.51760 (9)	0.64251 (18)	0.59890 (5)	0.0186 (2)	
C8	0.46876 (9)	0.40922 (18)	0.59144 (5)	0.0207 (2)	
H8A	0.5321	0.2974	0.5957	0.025*	
H8B	0.4262	0.3907	0.5480	0.025*	
C9	0.38662 (9)	0.38380 (19)	0.64646 (5)	0.0208 (2)	
C10	0.63667 (9)	0.67685 (19)	0.62955 (5)	0.0202 (2)	
C11	0.66216 (10)	0.8679 (2)	0.66689 (6)	0.0236 (2)	
H11	0.6031	0.9756	0.6727	0.028*	
C12	0.77389 (10)	0.9006 (2)	0.69558 (6)	0.0289 (3)	
H12	0.7907	1.0302	0.7212	0.035*	
C13	0.86102 (10)	0.7447 (3)	0.68696 (6)	0.0311 (3)	
H13	0.9370	0.7668	0.7070	0.037*	
C14	0.83690 (10)	0.5572 (2)	0.64908 (7)	0.0323 (3)	
H14	0.8969	0.4524	0.6423	0.039*	
C15	0.72483 (10)	0.5216 (2)	0.62083 (6)	0.0278 (3)	
H15	0.7084	0.3912	0.5955	0.033*	
C16	0.20572 (10)	0.5022 (2)	0.69194 (5)	0.0252 (2)	
H16A	0.1603	0.6424	0.6907	0.030*	
H16B	0.2522	0.4979	0.7345	0.030*	
C17	0.12199 (10)	0.3107 (2)	0.68985 (6)	0.0289 (3)	
H17	0.0687	0.3046	0.7236	0.035*	
C18	0.11499 (11)	0.1503 (2)	0.64588 (7)	0.0343 (3)	
H18A	0.1664	0.1488	0.6111	0.041*	
H18B	0.0585	0.0357	0.6489	0.041*	

### Atomic displacement parameters (Å^2^)

**Table:**

	*U*^11^	*U*^22^	*U*^33^	*U*^12^	*U*^13^	*U*^23^
O1	0.0268 (4)	0.0368 (5)	0.0297 (4)	0.0035 (4)	0.0000 (3)	0.0133 (4)
N1	0.0167 (4)	0.0233 (5)	0.0195 (4)	0.0001 (3)	0.0013 (3)	0.0025 (3)
N2	0.0174 (4)	0.0204 (5)	0.0228 (4)	−0.0012 (3)	−0.0004 (3)	0.0008 (3)
C1	0.0182 (4)	0.0190 (5)	0.0199 (5)	0.0011 (4)	−0.0008 (4)	−0.0002 (4)
C2	0.0184 (5)	0.0245 (6)	0.0272 (5)	−0.0004 (4)	−0.0013 (4)	0.0006 (4)
C3	0.0217 (5)	0.0290 (6)	0.0282 (6)	0.0039 (4)	−0.0060 (4)	−0.0007 (5)
C4	0.0282 (5)	0.0277 (6)	0.0230 (5)	0.0065 (4)	−0.0026 (4)	0.0034 (4)
C5	0.0254 (5)	0.0213 (5)	0.0239 (5)	0.0013 (4)	0.0010 (4)	0.0029 (4)
C6	0.0184 (4)	0.0189 (5)	0.0208 (5)	0.0013 (4)	−0.0007 (4)	−0.0007 (4)
C7	0.0175 (4)	0.0196 (5)	0.0188 (4)	−0.0006 (4)	0.0028 (3)	−0.0006 (4)
C8	0.0194 (5)	0.0184 (5)	0.0244 (5)	0.0001 (4)	0.0024 (4)	−0.0006 (4)
C9	0.0183 (5)	0.0203 (5)	0.0235 (5)	−0.0022 (4)	−0.0011 (4)	0.0019 (4)
C10	0.0175 (4)	0.0231 (5)	0.0200 (5)	0.0003 (4)	0.0011 (4)	0.0015 (4)
C11	0.0225 (5)	0.0236 (6)	0.0245 (5)	−0.0011 (4)	0.0002 (4)	−0.0002 (4)
C12	0.0269 (6)	0.0338 (7)	0.0254 (5)	−0.0077 (5)	−0.0030 (4)	−0.0013 (5)
C13	0.0187 (5)	0.0456 (8)	0.0286 (6)	−0.0047 (5)	−0.0028 (4)	0.0060 (5)
C14	0.0184 (5)	0.0401 (7)	0.0383 (7)	0.0056 (5)	0.0009 (4)	0.0014 (6)
C15	0.0208 (5)	0.0300 (6)	0.0326 (6)	0.0041 (4)	0.0010 (4)	−0.0045 (5)
C16	0.0242 (5)	0.0317 (6)	0.0202 (5)	0.0013 (4)	0.0045 (4)	−0.0019 (4)
C17	0.0203 (5)	0.0373 (7)	0.0294 (6)	0.0004 (5)	0.0044 (4)	0.0088 (5)
C18	0.0283 (6)	0.0298 (7)	0.0447 (7)	−0.0052 (5)	0.0009 (5)	0.0063 (6)

### Geometric parameters (Å, °)

**Table:**

O1—C9	1.2176 (14)	C8—H8B	0.9900
N1—C9	1.3713 (14)	C10—C15	1.3956 (15)
N1—C1	1.4207 (14)	C10—C11	1.3975 (16)
N1—C16	1.4725 (13)	C11—C12	1.3910 (15)
N2—C7	1.2884 (14)	C11—H11	0.9500
N2—C6	1.4041 (13)	C12—C13	1.3898 (19)
C1—C2	1.4038 (14)	C12—H12	0.9500
C1—C6	1.4100 (15)	C13—C14	1.383 (2)
C2—C3	1.3841 (16)	C13—H13	0.9500
C2—H2	0.9500	C14—C15	1.3938 (16)
C3—C4	1.3918 (18)	C14—H14	0.9500
C3—H3	0.9500	C15—H15	0.9500
C4—C5	1.3823 (16)	C16—C17	1.4981 (18)
C4—H4	0.9500	C16—H16A	0.9900
C5—C6	1.4040 (15)	C16—H16B	0.9900
C5—H5	0.9500	C17—C18	1.314 (2)
C7—C10	1.4824 (14)	C17—H17	0.9500
C7—C8	1.5133 (15)	C18—H18A	0.9500
C8—C9	1.5164 (15)	C18—H18B	0.9500
C8—H8A	0.9900		
			
C9—N1—C1	123.83 (9)	O1—C9—C8	122.78 (10)
C9—N1—C16	117.57 (9)	N1—C9—C8	114.45 (9)
C1—N1—C16	118.58 (9)	C15—C10—C11	119.22 (10)
C7—N2—C6	120.05 (10)	C15—C10—C7	120.75 (10)
C2—C1—C6	118.97 (10)	C11—C10—C7	120.02 (10)
C2—C1—N1	118.43 (9)	C12—C11—C10	120.11 (11)
C6—C1—N1	122.46 (9)	C12—C11—H11	119.9
C3—C2—C1	121.10 (11)	C10—C11—H11	119.9
C3—C2—H2	119.4	C13—C12—C11	120.31 (12)
C1—C2—H2	119.4	C13—C12—H12	119.8
C2—C3—C4	120.08 (10)	C11—C12—H12	119.8
C2—C3—H3	120.0	C14—C13—C12	119.86 (11)
C4—C3—H3	120.0	C14—C13—H13	120.1
C5—C4—C3	119.50 (11)	C12—C13—H13	120.1
C5—C4—H4	120.2	C13—C14—C15	120.22 (12)
C3—C4—H4	120.2	C13—C14—H14	119.9
C4—C5—C6	121.54 (11)	C15—C14—H14	119.9
C4—C5—H5	119.2	C14—C15—C10	120.26 (12)
C6—C5—H5	119.2	C14—C15—H15	119.9
N2—C6—C5	116.21 (10)	C10—C15—H15	119.9
N2—C6—C1	124.85 (9)	N1—C16—C17	115.57 (10)
C5—C6—C1	118.79 (10)	N1—C16—H16A	108.4
N2—C7—C10	118.19 (10)	C17—C16—H16A	108.4
N2—C7—C8	121.90 (9)	N1—C16—H16B	108.4
C10—C7—C8	119.86 (9)	C17—C16—H16B	108.4
C7—C8—C9	105.22 (9)	H16A—C16—H16B	107.4
C7—C8—H8A	110.7	C18—C17—C16	126.47 (11)
C9—C8—H8A	110.7	C18—C17—H17	116.8
C7—C8—H8B	110.7	C16—C17—H17	116.8
C9—C8—H8B	110.7	C17—C18—H18A	120.0
H8A—C8—H8B	108.8	C17—C18—H18B	120.0
O1—C9—N1	122.69 (10)	H18A—C18—H18B	120.0
			
C9—N1—C1—C2	138.75 (11)	C1—N1—C9—O1	178.41 (11)
C16—N1—C1—C2	−42.98 (14)	C16—N1—C9—O1	0.11 (17)
C9—N1—C1—C6	−45.56 (16)	C1—N1—C9—C8	1.68 (15)
C16—N1—C1—C6	132.71 (11)	C16—N1—C9—C8	−176.62 (9)
C6—C1—C2—C3	0.28 (17)	C7—C8—C9—O1	−106.66 (12)
N1—C1—C2—C3	176.12 (11)	C7—C8—C9—N1	70.06 (12)
C1—C2—C3—C4	−0.74 (18)	N2—C7—C10—C15	−148.72 (11)
C2—C3—C4—C5	0.15 (18)	C8—C7—C10—C15	33.95 (15)
C3—C4—C5—C6	0.91 (18)	N2—C7—C10—C11	30.35 (15)
C7—N2—C6—C5	−142.10 (11)	C8—C7—C10—C11	−146.97 (11)
C7—N2—C6—C1	42.31 (15)	C15—C10—C11—C12	−0.79 (17)
C4—C5—C6—N2	−177.22 (10)	C7—C10—C11—C12	−179.88 (10)
C4—C5—C6—C1	−1.36 (17)	C10—C11—C12—C13	0.45 (18)
C2—C1—C6—N2	176.23 (10)	C11—C12—C13—C14	0.70 (19)
N1—C1—C6—N2	0.56 (17)	C12—C13—C14—C15	−1.5 (2)
C2—C1—C6—C5	0.75 (16)	C13—C14—C15—C10	1.2 (2)
N1—C1—C6—C5	−174.92 (10)	C11—C10—C15—C14	−0.01 (18)
C6—N2—C7—C10	−175.11 (9)	C7—C10—C15—C14	179.07 (11)
C6—N2—C7—C8	2.15 (15)	C9—N1—C16—C17	−83.98 (13)
N2—C7—C8—C9	−75.36 (12)	C1—N1—C16—C17	97.64 (12)
C10—C7—C8—C9	101.86 (10)	N1—C16—C17—C18	0.49 (18)

## References

[bb1] Ahabchane, A. H., Keita, A. & Essassi, E. M. (1999). *C. R. Ser. IIC*, **2**, 519–523.

[bb2] Barbour, L. J. (2001). *J. Supramol. Chem.* **1**, 189–191.

[bb3] Bruker (2008). *APEX2* and *SAINT* Bruker AXS Inc., Madison, Wisconsin, USA.

[bb4] Sheldrick, G. M. (2008). *Acta Cryst.* A**64**, 112–122. 10.1107/S0108767307043930 18156677

[bb5] Westrip, S. P. (2010). *J. Appl. Cryst.* **43** Submitted.

